# Focal stimulation of the temporoparietal junction improves rationality in prosocial decision-making

**DOI:** 10.1038/s41598-020-76956-9

**Published:** 2020-11-20

**Authors:** Flora Li, Sheryl Ball, Xiaomeng Zhang, Alec Smith

**Affiliations:** 1 Economics Experimental Lab, Nanjing Audit University, Nanjing, China; 2 Department of Economics, Virginia Tech, Blacksburg, VA USA; 3 School of Neuroscience, Virginia Tech, Blacksburg, VA USA

**Keywords:** Social behaviour, Social neuroscience, Human behaviour, Neuroscience, Neurophysiology

## Abstract

We tested the hypothesis that modulation of neurocomputational inputs to value-based decision-making affects the rationality of economic choices. The brain’s right temporoparietal junction (rTPJ) has been functionally associated with both social behavior and with domain-general information processing and attention. To identify the causal function of rTPJ in prosocial decisions, we administered focal high definition transcranial direct current stimulation (HD-tDCS) while participants allocated money between themselves and a charity in a modified dictator game. Anodal stimulation led to improved rationality as well as increased charitable giving and egalitarianism, resulting in more consistent and efficient choices and increased sensitivity to the price of giving. These results are consistent with the theory that anodal stimulation of the rTPJ increases the precision of value computations in social decision-making. Our results demonstrate that theories of rTPJ function should account for the multifaceted role of the rTPJ in the representation of social inputs into value-based decisions.

## Introduction

Rational choice theory assumes that people’s preferences are stable and that they obey normative axioms, which typically require that decision-makers prefer more to less, that they effortlessly select their preferred alternative, and that there are no cycles in preferences. The bridge between these normative axioms and actual choices is the theory of revealed preference^1–3^. This approach equates rational choice with consistency: a decision-maker who chooses an apple over a banana must not instead select the banana when a cherry is added to the set of available options. The rational choice approach generates a mathematically tractable model of decision-making that is widely used in economics and related disciplines.

However, actual choice behavior often does violate normative axioms, an observation that led to the development of descriptive, behavioral models of decision-making^4–6^. Choice inconsistencies may result from bounded rationality^7^, the use of heuristics^8^, or limited attention^9,10^. More recently, neuroimaging studies demonstrate that value-based decisions in both economic and social contexts result from neural computations that involve representing, evaluating, and selecting options from a choice set^11,12^. These computations rely upon the brain valuation system, a functional network that includes the ventral striatum and ventromedial prefrontal cortex^13–15^, and that receives valuation input from other brain regions including the dorsolateral prefrontal cortex^16^ and the temporoparietal junction (TPJ)^17,18^. The computation and integration of decision values is instantiated by neurons whose firing rates are inherently noisy, and this inherent stochasticity may be the underlying cause of choice inconsistencies^18–22^. This neurocomputational framework suggests that interventions that manipulate the action potential of neurons involved in value computations may affect the precision of these computations and, therefore, the consistency and rationality of choices.

We hypothesized that in the context of social decisions, modulation of neural activity in the brain’s right TPJ (rTPJ) would affect the precision of social value representations. We tested this hypothesis in the context of charitable giving through focal noninvasive brain stimulation (NBS). NBS is a neuromodulation technique used to identify the causal effect of changing cortical excitability on cognition and behavior^23–25^. While many NBS studies of TPJ^26–30^ focus on social cognition and behavior, this region also performs other, domain-general functions, including integrating and processing sensory information and regulating attention^31–38^. Because resource constraints on these functions can result in choice inconsistencies and seemingly irrational behavior^21,22,39^, it is critical to simultaneously measure the effect of TPJ-NBS on both revealed social preferences and choice quality.

**Figure 1:**
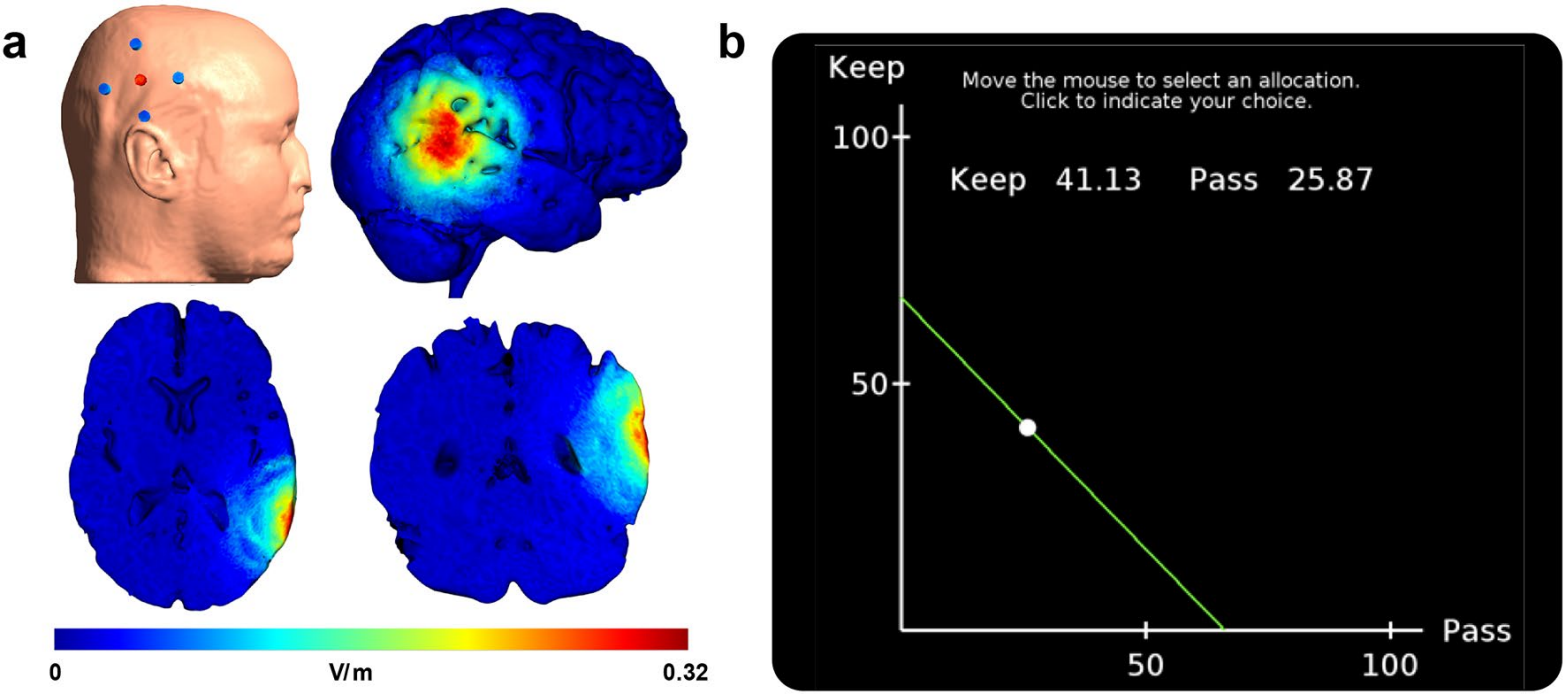
HD-tDCS current modeling and decision task. (**a**) Current modeling simulates the norm of the electric field (V/m) induced by anodal stimulation over rTPJ, and shows the stimulation pattern on the surface of the brain with a sagittal view, and the depth of stimulation with an axial and a coronal slice. (**b**) Experimental task, where participants choose an allocation on or under the budget line (green line) during 50 independent trials. Points on (efficient) and under (inefficient) the line represents all feasible allocations of tokens.

In this experiment, human participants allocated money between themselves and a charity in a modified dictator game^22,40–45^ with real financial incentives while undergoing either anodal, cathodal, or sham high-definition transcranial direct current stimulation (HD-tDCS) over the rTPJ (Fig. 1a). HD-tDCS employs an array of small electrodes to focus electrical current stimulation over a specific region of the brain^46,47^, and computational models of electrical field strength demonstrate the improved precision of HD-tDCS arrays relative to conventional montages^48–50^. To determine the effect of stimulation on both decision quality and preferences for giving, participants made decisions from 50 budget sets (Fig. 1b) that differed both in the amount of money to be allocated and the price of giving to the charity. The variation of prices and endowments makes it possible to evaluate the extent to which participants behaved rationally, in the sense that their choices are consistent with utility maximization. This novel combination of an economic paradigm that measures choice quality with focal neuromodulation makes it possible to simultaneously measure the effect of stimulation on both prosocial behavior and economic rationality.

We find that HD-tDCS over rTPJ causally affects the quality of social decisions as well as individuals’ social preferences. Participants who received anodal stimulation made choices that were more consistent with economic rationality, that were more responsive to prices, and that resulted in improved total welfare relative to the other treatments. Furthermore, anodal stimulation led to both increased donations to the charity and more relatively equal splits of available funds than sham or cathodal stimulation, consistent with imaging and NBS studies suggesting a role for TPJ in egalitarianism^51,52^. Thus, focal anodal stimulation over rTPJ simultaneously resulted in both higher quality and more fair-minded social decisions. These results support the theory that TPJ employs neurocomputational resources to construct value representations in social decision-making^17^ and that HD-tDCS over rTPJ may help remedy deficiencies in social function.

## Results

### Anodal HD-tDCS improves choice quality and rationality

**Figure 2:**
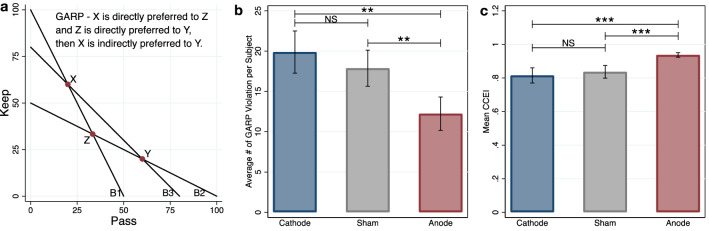
GARP illustration and results. *P < .1, **P < .05, ***P < .01. Data in mean ± SEM. (**a**) GARP implies that if an individual chooses X over Z when the budget line is B1, and Z over Y when the budget line is B2, she cannot choose Y over X when the budget line is B3. (**b**) Subject level measurements of GARP violations show that anodal participants have the fewest violations. A one-sided t-test of the difference in mean number of GARP violations (anode vs. cathode) rejects the null hypothesis of no difference (*P* = .0126). (**c**) Subject level CCEI across treatments. CCEI measures the severity of GARP violations. Violations of GARP are the least severe in the anodal treatment; a one-sided t-test of the difference in mean CCEI (anode vs. cathode) rejects the null hypothesis of no difference (*P* = .0059).

For utility theory to hold several choice axioms must be satisfied. Therefore, for each treatment condition we computed both the frequency and severity of violations of (1) the Monotonicity Axiom (Monotonicity), (2) the Generalized Axiom of Revealed Preference (GARP)^22,40,43,45^, and 3) the Weak Axiom of Revealed Preference (WARP)^53,54^. Monotonicity is the most straightforward axiom, requiring that individuals prefer more tokens to less; in our design, this implies that choices must be along the budget line rather than in the interior of the choice set (Supplementary Fig. 5a). GARP requires that preferences are transitive; that is, they do not exhibit choice cycles (Fig. 2a). WARP requires that choices are consistent in that whenever one allocation is chosen over another, the participant must reveal the same preferences when choosing from other budget sets where these options are both available (Supplementary Fig. 6a).

Figure 2b shows that the fewest GARP violations occurred in the anodal condition (anode: mean = 12.24, 95% CI = 8.01–16.46; cathode: mean = 19.88, 95% CI = 14.57–25.20; sham: mean = 17.88, 95% CI = 13.33–22.44; one-sided t-test anode < cathode: *t*_66_ = 2.29, *P* = 0.0126, Cohen's *d* = 0.56; anode < sham: *t*_66_ = 1.85, *P* = 0.0345, *d* = 0.45), while cathodal and sham conditions are not different from each other (two-sided t-test: *t*_66_ = 0.58, *P* = 0.5630, *d* = 0.14).

We observe a similar pattern for the other choice axioms (Supplementary Figs. 5b and 6b). In essence, the fewest violations of rationality occur in the anodal condition, and the most violations occur in the cathodal condition (Monotonicity: anode mean = 2.12, 95% CI = 0.35–3.89, cathode mean = 4.44, 95% CI = 1.28–7.60, one-sided t-test: *t*_66_ = 1.31, *P* = 0.0981, *d* = 0.32; WARP: anode mean = 8.76, 95% CI = 6.10–11.43, cathode mean = 13.41, 95% CI = 9.88–16.94, one-sided t-test: *t*_66_ = 2.14, *P* = 0.0182, *d* = 0.52). We also observe that, similar to the GARP result, the cathodal condition is not different from sham for either Monotonicity or WARP (Monotonicity: sham mean = 2.97, 95% CI = 0.98–4.96, two-sided t-test: *t*_66_ = 0.80, *P* = 0.4257, *d* = 0.19; WARP: sham mean = 13.00, 95% CI = 9.80–16.20, two-sided t-test: *t*_66_ = 0.18, *P* = 0.8611, *d* = 0.04).

GARP and WARP violations identify choice inconsistencies but are insensitive to the magnitude of the violation. A precise measure of how close choices are to satisfying GARP is Afriat’s Critical Cost Efficiency Index (CCEI, Supplementary Fig. 4)^40,44,53^. This index reflects the extent to which each participant’s budget sets must be shifted outwards to be consistent with utility maximization; choices that are more consistent with GARP result in CCEI values that are closer to 1. We find that the mean CCEI in the anodal condition is significantly higher than in both the cathodal and sham conditions (anode: mean = 0.94; 95% CI = 0.91–0.96; cathode: mean = 0.82; 95% CI = 0.72–0.91; sham: mean = 0.84, 95% CI = 0.76–0.91; one-sided t-test anode < cathode: *t*_66_ = 2.59, *P* = 0.0059, *d* = 0.63; anode < sham: *t*_66_ = 2.50, *P* = 0.0075, *d* = 0.61), but that the means of the CCEI in the sham and cathode conditions are not different from each other (two-sided t-test: *t*_66_ = 0.37, *P* = 0.7155, *d* = 0.09). This consistent pattern of decreased frequency and severity of rationality violations demonstrates that focal anodal tDCS over rTPJ causes individuals to make more economically rational decisions in the charitable giving task.

### Anodal HD-tDCS enhances charitable giving and egalitarianism

**Figure 3:**
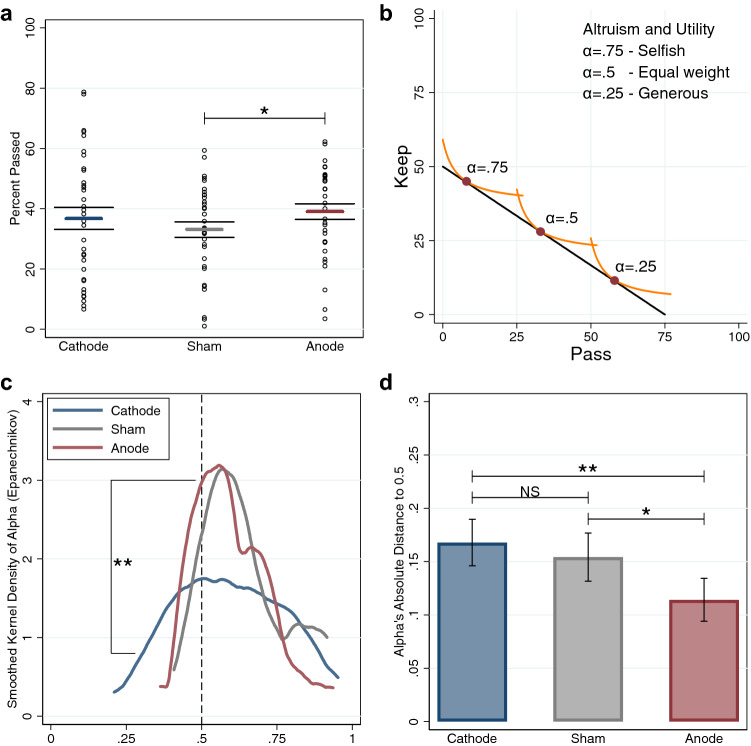
CES utility and alpha parameter. *P < .1, **P < .05, ***P < .01. Data in mean ± SEM. (**a**) Subject level percent passed box plot with colored dots representing each participant. A one-sided t-test shows anodal participants give more than sham participants (*P* = .0540). A variance comparison test for equality of standard deviations shows that the anodal distribution is the most concentrated and the cathodal distribution is the least concentrated (*P* = .0275). (**b**) Example of CES Utility Functions, where *α* is the weight on one’s own payoff and 1 − *α* is the weight on money for the charity. Three hypothetical individuals with different preferences *α* = 0.75 (selfish), 0.5 (equal weight for self and other), and 0.25 (generous) are illustrated. (**c**) Smoothed kernel density shows that anodal stimulation results in a concentration of *α* values over 0.5; a one-sided variance comparison test for equality of standard deviations (anode vs. cathode) rejects the null hypothesis of no difference (*P* = .0192). (**d**) Subject level absolute distance from *α* to 0.5 shows that *α* is closer to 0.5 in the anodal than in the cathodal treatment; a one-sided t-test of the difference in means (anode vs. cathode) rejects the null hypothesis of no difference (*P* = .0374).

To study the effect of stimulation on prosocial behavior, we first evaluate each individual’s giving behavior by computing the percent passed to the charity (${\pi }_{p}/P$) each trial, where ${\pi }_{p}$ is the number of tokens passed out of a maximum of *P* tokens. When this ratio equals 1(0) an individual has made a completely altruistic (selfish) choice. Sham participants, on average, passed 33.11% of the total tokens, consistent with previous work using this task^41,44^. Figure 3a shows that individuals’ average percent passed in the anodal treatment is marginally higher than in the sham treatment (anode: mean = 0.39, 95% CI = 0.34–0.44; sham: mean = 0.33, 95% CI = 0.28–0.38; one-sided t-test: *t*_66_ = 1.63, *P* = 0.0540, *d* = 0.40), while the cathodal treatment is not different from either anode or sham (cathode: mean = 0.37, 95% CI = 0.29–0.44; two-sided t-test cathode vs. anode: *t*_66_ = 0.52, *P* = 0.6021, *d* = 0.13; cathode vs. sham: *t*_66_ = 0.81, *P* = 0.4187, *d* = 0.20).

While the sample mean captures the central tendency of the data, distributional analysis helps understand its variability. Figure 3a shows that the anodal and cathodal distributions of percent passed are also significantly different (Epps-Singleton distribution (ES) test: *P* = 0.0429). Behavior in the anodal treatment is concentrated around the equal split, while results from the cathodal treatment are spread out; statistically, the standard deviation of percent passed in the cathodal treatment is significantly larger than in the anodal treatment (anode: SD = 0.150; cathode: SD = 0.211; one-sided Variance Comparison (VC) test: *F*_33,33_ = 1.97, *P* = 0.0275).

We performed additional analysis of preferences across treatments by estimating individual preferences using a parametric structural model. Because linear utility models generate the prediction that participants will either keep all of their tokens or pass all of their tokens, we employed a constant elasticity of substitution (CES) utility function to measure preferences (see “Methods”)^40,41,43,44^. The estimated parameter *α* gives the utility weight each participant places on the payment to the charity, where *α* = 1 is completely selfish, *α* = 0.5 is egalitarian, and *α* = 0 is completely selfless (Fig. 3b).

Analysis of *α* by treatment confirms the results of the above analysis of percent passed. The mean estimate of the parameter *α* does not differ across three treatments (anode: mean = 0.60, 95% CI = 0.55–0.64; cathode: mean = 0.60, 95% CI = 0.53–0.66; sham: mean = 0.65, 95% CI = 0.60–0.70; one-sided t-test anode < cathode: *t*_66_ = 0.07, *P* = 0.5263, *d* = 0.02; anode < sham: *t*_66_ = 1.45, *P* = 0.0761, *d* = 0.35; sham < cathode: *t*_66_ = 1.25, *P* = 0.8915, *d* = 0.30). Similar to the distributional results for the percent passed, we find that both the distribution shape and standard deviation of *α* are different (Fig. 3c). More anodal individuals have $\alpha$ close to 0.5, and fewer have *α* close to either 0 or 1. The anodal distribution of *α* is slim, bell-shaped, and tightly concentrated around 0.5, whereas the cathodal distribution looks more like a uniform distribution (ES test: *P* = 0.0885). The distribution of *α* in the sham condition is also different from the cathodal condition, but not from the anodal condition (ES test: cathode vs. sham *P* = 0.0938, anode vs. sham *P* = 0.5768). A VC test shows that the anodal distribution is more concentrated than the cathodal distribution (anode: SD = 0.022; cathode: SD = 0.032; one-sided VC test: *F*_33,33_ = 2.08, *P* = 0.0192). To test whether anodal stimulation over rTPJ induces egalitarianism, we compute the absolute distance between each individual’s estimated *α* and 0.5 and compare the resulting differences across treatments. Figure 3d shows that, on average, anodal participants have *α* closer to 0.5 than cathodal participants (anode: mean = 0.11, 95% CI = 0.07–0.15; cathode: mean = 0.17, 95% CI = 0.12–0.21; one-sided t-test: *t*_66_ = 1.81, *P* = 0.0374, *d* = 0.44).

### Anodal HD-tDCS increases price sensitivity and elasticity

**Figure 4:**
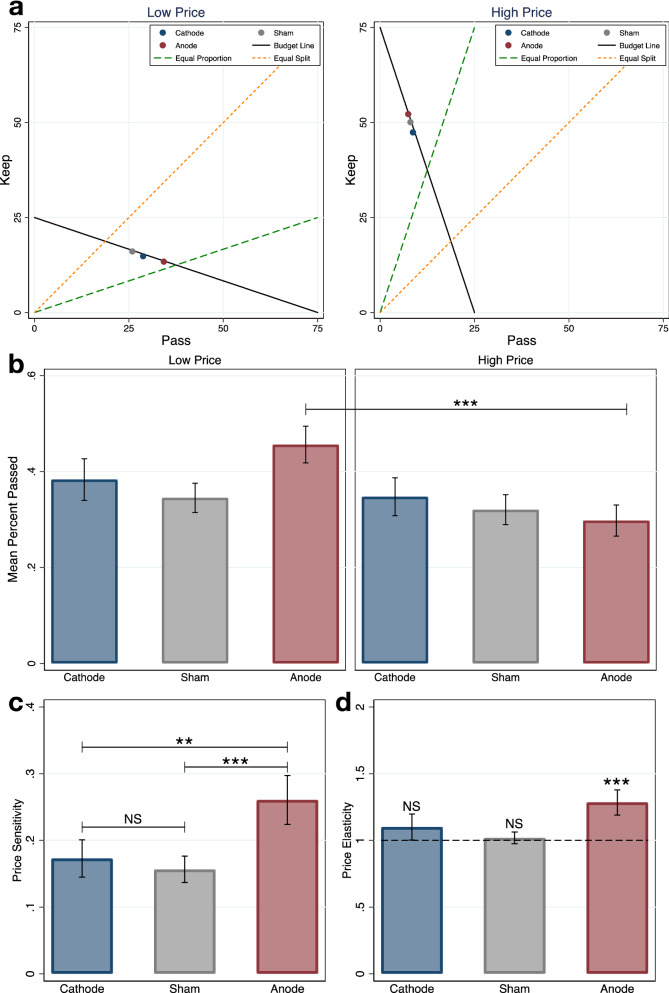
Price sensitivity and elasticity. *P < .1, **P < .05, ***P < .01. Data in mean ± SEM. (**a**) Stylized illustration of price sensitivity in the experimental task. Subject level mean percent kept and percent passed are used in the diagram. Anodal participants exhibit the most shift in choice respect to price change. Anodal participants’ choices are closest to equal proportion when the price is low. (**b**) Subject level percent passed shows only anodal participants’ behavior changes with prices; a one-sided t-test of the difference in means (high vs. low price) rejects the null hypothesis of no difference (*P* = .0012). (**c**) Subject level price sensitivity indicates that anodal participants are the most sensitive to price change; one-sided t-tests of the difference in means rejects the null hypothesis of no difference (anode > cathode, *P* = .0306; anode > sham, *P* = .0073). (**d**) Subject level price elasticity shows that anodal participants are elastic in price, whereas cathodal and sham participants are not different from unit elastic; a one-sided t-test rejects the null hypothesis of anodal price elasticity = 1 (*P* = .0025).

The decisions of rational individuals who are not entirely selfish or selfless should be price sensitive. For example, individuals may keep fewer tokens for themselves as the relative price of tokens for the charity decreases. Figure 4a plots the mean allocation choices by treatment for both low price (slope = 1/3) and high price (slope = 3) budget lines. The strongest response to price changes occurs in the anodal treatment where average choices are closest to equal proportion when prices are low and farthest away when prices are high. The above findings are confirmed statistically when price sensitivity is measured as the absolute change in mean percent tokens passed from low price (budget slope < 1) to high price (budget slope > 1) (Fig. 4b). In our study token allocation decisions are responsive to price changes in the anodal treatment (anodal percent passed in the high price: mean = 0.30, 95% CI = 0.23–0.36; low price: mean = 0.46, 95% CI = 0.38–0.53; one-sided t-test high price < low price: *t*_66_ = 3.16, *P* = 0.0012, *d* = 0.77), but not in the other two treatments (cathodal high price: mean = 0.35, 95% CI = 0.27–0.43; low price: mean = 0.38, 95% CI = 0.30–0.47; sham high price: mean = 0.32, 95% CI = 0.26–0.38; low price: mean = 0.35, 95% CI = 0.28–0.41; two-sided t-test cathode high vs. low price: *t*_66_ = 0.61, *P* = 0.5433, *d* = 0.15; sham high vs. low price: *t*_66_ = 0.56, *P* = 0.5743, *d* = 0.14). In addition, only anodal participants’ percent passed at low prices is insignificantly different from 0.5, the equal proportion split (two-sided t-test: *t*_33_ = 1.13, *P* = 0.2648, *d* = 0.19), demonstrating increased egalitarianism in anodal participants when the price of giving to the charity is low.

Figure 4c compares price sensitivity directly across treatments. Anodal participants are the most price sensitive (anode: mean = 0.26, 95% CI = 0.19–0.34; cathode: mean = 0.17, 95% CI = 0.12–0.23; sham: mean = 0.16, 95% CI = 0.12–0.20; one-sided t-test anode > cathode: *t*_66_ = 1.90, *P* = 0.0306, *d* = 0.46; anode > sham: *t*_66_ = 2.51, *P* = 0.0073, *d* = 0.61), whereas participants in cathode and sham are equally sensitive to price changes (two-sided t-test cathode vs. sham: *t*_66_ = 0.48, *P* = 0.6362, *d* = 0.12).

An additional measure of price sensitivity commonly used in economics is the price elasticity of demand, the ratio of the percentage change in quantity demanded to the percentage change in price. Figure 4d shows the price elasticity of demand for tokens for self across treatments. Elasticity in the anodal condition is significantly greater than 1 (mean = 1.28, 95% CI = 1.09–1.48, one–sided t-test: *t*_33_ = 3.00, *P* = 0.0025, *d* = 0.52) indicating that demand is elastic, which means that quantity changes outpace price changes. Conversely, computed cathodal and sham elasticities are not statistically different from 1 (cathode: mean = 1.10, 95% CI = 0.90–1.30, two-sided t-test: *t*_33_ = 1.03, *P* = 0.3104, *d* = 0.18; sham: mean = 1.02, 95% CI = 0.93–1.11, two-sided t-test: *t*_33_ = 0.44, *P* = 0.6611, *d* = 0.08) indicating unit elasticity where quantity changes match changes in price. These results further demonstrate that participants in the anodal condition were more responsive to stimulus-driven changes in the cost (in terms of tokens kept) of donating to the charity.

## Discussion

We find that focal anodal stimulation over the rTPJ results in improved choice quality in the form of fewer monotonicity violations, increased consistency with a well-defined objective function—which is required for choices to be economically rational—and greater price sensitivity. Anodal stimulation also results in more prosocial behavior that is concentrated around an egalitarian social norm. Our results are consistent with the notion that anodal stimulation of the rTPJ increases the precision of value computations in the charitable giving task. Making decisions in the task involves computing representations of the value of different allocations of tokens to the self and an abstract other (the charity). The relative value of giving to the charity versus keeping money for oneself is clearly influenced by economic considerations, including the endowment of tokens and the price of giving. More generally, the decision may be simultaneously determined by a number of factors, including considerations about equity and efficiency, empathy for others (e.g., the beneficiaries of donations to the food bank), and considerations about one’s social or self-image. The brain then combines these inputs into representations of the value of the available options. The effect of stimulation on both preferences for giving and the quality of choices is consistent with the notion that the rTPJ recruits domain-general computational resources to generate value representations in social decisions. In general, HD-tDCS over rTPJ is likely to generate small changes in the spike timing and firing rates of neurons in this region that result in increased population coherence^55–57^. We conjecture that the effect of anodal stimulation is to make rTPJ more responsive to sensory inputs, focusing attention on the task and increasing the precision of social value computations^58,59^.

This claim has a basis in both the economics and neuroscience literatures. Work in economics on bounded rationality examines how limited attention could impact rationality^10,60.^ Neuroscience research establishes the importance of TPJ in attention allocation and information processing^31–38^. Together, these results provide a plausible link between TPJ’s function in information processing and economic rationality due to attention shifting. Our result that anodal HD-tDCS over rTPJ causes improved economic rationality and choice quality in social decisions provides additional evidence in support of this link.

A prominent theory states that the mechanism by which tDCS affects brain function is polarity dependent, so anodal tDCS increases the neural excitability of the stimulated region shifting the resting membrane potential closer to the depolarization threshold and resulting in a higher spiking rate, while cathodal tDCS has the opposite effect^61,62^. While for some analyses we find that cathodal stimulation has the opposite effect on prosocial behavior from anodal stimulation, we also find that the some effects of cathodal stimulation are no different from sham. These inconsistent effects of cathodal stimulation on decision making parallel research on the motor cortex that finds cathodal stimulation may have the opposite effect of anodal^61^, the same effect^63^, or no effect at all^64^. Similar inconsistencies have been found in NBS studies of visual, auditory, and somatosensory function^65^ and cognitive and emotional processing^66^. In the future, new current models that incorporate detailed information about brain structure may shed light on the polarity-dependent effect of stimulation.

HD-tDCS’s effectiveness^46^, minimal side effects^47^, and cost-effectiveness have prompted research on potential therapeutic uses^67–69^. Our findings demonstrate that anodal HD-tDCS over rTPJ improves choice quality and enhances social decisions. In addition to the present study of healthy adults, our study suggests that clinical use of focal NBS over rTPJ may be effective for managing or treating disorders associated with impaired rationality or social cognition.

## Methods

### Transcranial direct current stimulation

We used a neuroConn DC-Stimulator MC (München, Germany) to administer the stimulation. During the behavioral task, participants received either anodal, cathodal, or sham HD-tDCS. Stimulation was delivered over the right temporoparietal junction (CP6 on a 10/10 measurement system) with an intensity of 2 mA using a 4 × 1 ring electrode montage, with a 15 s ramp-up at the start of stimulation and a 15 s ramp-down at the conclusion. The tDCS stimulation was turned on after participants read the instructions and completed three practice trials. Then participants were required to wait for 2 min to begin the behavioral task to ensure that they were comfortable and allow the effects of the stimulation to equilibrate. In the anodal and cathodal conditions, participants made decisions while receiving stimulation. In the sham condition, participants received 30 s of stimulation at the start of the 2-min wait time in order to blind them to the treatment, but did not receive stimulation while making decisions. Simulation of the anodal stimulation is achieved using current modeling software (Soterix and SimNibs) to indicate the precise stimulation location.

Participants completed the task at their own pace, and stimulation was terminated when participants finished the task. The average stimulation duration was 11.79 min, which is well within the safety limit of tDCS best practices^70,71^. We recruited 109 healthy participants from the university community. We analyzed data from 102 participants (age: max 65, min 18, mean 22.64, SD 7.19; gender: 55 males, 47 females). Three participants discontinued participation after electrodes were applied: One participant discontinued due to an adverse event^72^, and two participants discontinued due to discomfort from tDCS. In addition, three participants discontinued participation prior to electrode placement: one participant reported feeling lightheaded when they arrived, one participant was excluded due to a scalp condition, and a third was unwilling to allow gel application in their hair. Data were excluded for one participant who completed the experiment because more conductive gel was applied than our protocol specified which might have interfered with the tDCS procedure. Out of 102 participants, 7 were university staff or faculty, 15 were graduate students, and the rest were undergraduate students. Of the 102 participants, 34 received anodal tDCS stimulation (age: max 47, min 18, mean 22.65, SD 5.75; gender: 17 males, 17 females), 34 received cathodal tDCS stimulation (age: max 53, min 18, mean 22.38, SD 7.00; gender: 21 males, 13 females), and 34 received sham tDCS stimulation (age: max 65, min 18, mean 22.88, SD 8.72; gender: 17 males, 17 females). Both the experimenter directly interacting with the participants during the task and the participants themselves were blind to their treatment group. A post-experiment questionnaire indicated that participants could not distinguish among different tDCS treatments (see Supplementary Fig. 3).

### Modified dictator game

The modified dictator game was programmed using MATLAB and Psychtoolbox^73^. This task is simple, flexible, and designed to encourage rational decisions. Similar task designs have been used in behavioral experiments to study decision quality, altruism, risk preferences, and political behavior^40,41, 43,44^. In the task, participants allocate an endowment of tokens between themselves and a local food bank, Feeding America Southwest Virginia (FASWVA), during 50 independent trials. In each trial, participants could choose allocations either on or under a graphical representation of a budget line (Fig. 1b). Keeping tokens for themselves was referred to as Keep and allocating tokens to FASWVA was referred to as Pass. The endowment and relative price of contributing to the charity were randomly varied across 50 trials, such that the budget line intersected with at least one axis at 50 or more tokens, with a maximum intercept value of 100 tokens^44^. After participants made each allocation, the feedback was shown on the next screen. Participants received $20 compensation. In addition, one trial was randomly selected for payment and both the participant and the charity received $0.50 for each token allocated by the participant in that trial.

### Other-regarding preferences

We fit each individual’s choice data with a parametric constant elasticity of substitution (CES) utility function that measures the extent of other-regarding behavior^40,44^.

$$u\left({\pi }_{k},{\pi }_{p}\right)={\left(\alpha {{\pi }_{k}}^{\rho }+\left(1-\alpha \right){{\pi }_{p}}^{\rho }\right)}^{1/\rho }$$

In the CES utility function, ${\pi }_{k}$ and ${\pi }_{p}$ represent tokens allocated to self and the charity respectively. The parameter $\alpha \in [\mathrm{0,1}]$ measures an individual’s other-regarding behavior, where $\alpha =0$ means complete selflessness, $\alpha =1$ means complete selfishness, and α = 0.5 means equal division (see Fig. 3b). The parameter $\rho \in [-\infty ,1]$ describes the curvature of an individual’s indifference curve and captures the tradeoff between maximizing the number of tokens distributed and fair division. When $\rho$ approaches 1, the indifference curve approaches a straight line, which represents the preferences of an individual who either keeps all of the tokens or gives them all to the charity. When $\rho$ approaches $-\infty$, the indifference curve approaches an L-shaped or Leontief indifference curve, and selected allocations occur closer to the center of the budget line.

The budget constraint is $\frac{{\pi }_{k}}{K}+\frac{{\pi }_{p}}{P}\le 1$, where *K* refers to the maximum possible tokens to self, and *P* refers to the maximum possible tokens to the charity. We then solved the expenditure function (see supplementary methods for detail):

$$\frac{{\pi }_{k}}{K}=\frac{{\left(\frac{\alpha }{1-\alpha }\right)}^{\frac{1}{1-\rho }}}{{\left(\frac{\alpha }{1-\alpha }\right)}^{\frac{1}{1-\rho }}+{\left(\frac{K}{P}\right)}^{\frac{\rho }{\rho -1}}}$$

For each treatment, we estimated a hierarchical random utility model (HRUM) with subject level random effects. The model allowed the parameters $\alpha$ and $\rho$ to vary across both tDCS treatments and individuals, and the variance of the residual was allowed to differ across the tDCS treatments. Here the subscript *i* represents the participant and the superscript *n* represents the trial.

$$\frac{{{\pi }_{k,i}}^{n}}{{K}_{i}^{n}}=\frac{{\left(\frac{{\alpha }_{i}}{1-{\alpha }_{i}}\right)}^{\frac{1}{1-{\rho }_{i}}}}{{\left(\frac{{\alpha }_{i}}{1-{\alpha }_{i}}\right)}^{\frac{1}{1-{\rho }_{i}}}+{\left(\frac{{K}_{i}^{n}}{{P}_{i}^{n}}\right)}^{\frac{{\rho }_{i}}{{\rho }_{i}-1}}}+{{\varepsilon }_{i}}^{n}$$

### Economic rationality

For decision bundles ${x}_{1}$, ${x}_{2}$, and ${x}_{3}$ we define:

$$\begin{aligned} & {\text{Directly}}\;{\text{Revealed}}\;{\text{Preference}}\left( { \succsim }^{D} \right): x_{1} { \succsim }^{D} x_{2} \; {\text{if}}\;x_{1} ,x_{2} \in B,\;{\text{and}}\;x_{1} \in C\left( B \right) \\ & {\text{Indirectly}}\;{\text{Revealed}}\;{\text{Preference}}\left( { \succsim }^{I} \right): x_{1} { \succsim }^{I} x_{2} \; {\text{if}}\;x_{1} {\succsim }^{D} x_{3} ,\;{\text{and}}\;x_{3} { \succsim }^{D} x_{2} , \\ \end{aligned}$$

where *C(B)* is the (possibly unique) set of bundles selected from budget set *B*. Monotonicity requires that, for ${x}_{1}$, ${x}_{2} \in B$, if ${x}_{1}\gg {x}_{2}$, then ${x}_{1}\succ {x}_{2}$. Utility maximization and Monotonicity require individuals to choose consumption bundles on the budget constraint. Any consumption bundle $x$ that is greater than 0.05 tokens away from the budget line was counted as a Monotonicity violation. The severity of Monotonicity violations was tested by calculating the absolute distance to the budget constraint: the greater the distance is, the more severe the violation is. Monotonicity implies that indifference curves are thin, downward sloping, and do not cross each other.

WARP requires that, if ${x}_{1}{\succsim }^{D}{x}_{2}$ then we do not have ${x}_{2}{\succsim }^{D}{x}_{1}$. If both consumption bundles ${x}_{1}, {x}_{2}$ are available for two different budget constraints B, B’, and an individual chooses ${x}_{1}$ under B and chooses ${x}_{2}$ under B’, then she violated WARP. WARP is necessary for the existence of a strictly convex utility function.

GARP requires that, if ${x}_{1}{\succsim }^{I}{x}_{2}$, then we do not have ${x}_{2}{\succsim }^{D}{x}_{1}$. Violation of Transitivity implies violation of GARP, therefore GARP rules out preference cycles. The severity of GARP violations is measured using Afriat’s (1972) Critical Cost Efficiency Index (CCEI)^53^, where CCEI $\in [\mathrm{0,1}]$. CCEI measures how far budget constraints must be shifted to avoid a GARP violation. CCEI = 1 represents no GARP violation, and CCEI = 0 represents the most severe GARP violation. GARP is necessary and sufficient for the existence of a well-behaved utility function.

### Price sensitivity and elasticity

Price sensitivity is denoted as the absolute change in percent passed comparing low vs. high prices. Assuming constant elasticity, price elasticity of tokens for self (*E*_*S*_) is

$$E_{S} = \frac{{\% \Delta \pi_{k} }}{{\% \Delta \frac{1}{K}}} = \frac{{\partial \pi_{k} }}{{\partial \frac{1}{K}}} \cdot \frac{\frac{1}{K}}{{\pi_{k} }} = \frac{{\partial \pi_{k} }}{{\partial \frac{1}{K}}} \cdot \frac{{\partial \ln \pi_{k} }}{{\partial \pi_{k} }} \cdot \frac{1}{K} = \frac{1}{K} \cdot \frac{{\partial \ln \pi_{k} }}{{\partial \frac{1}{K}}} = \frac{{\partial \frac{1}{K}}}{{\partial \ln \frac{1}{K}}} \cdot \frac{{\partial \ln \pi_{k} }}{{\partial \frac{1}{K}}} = \frac{{\partial \ln \pi_{k} }}{{\partial \ln \frac{1}{K}}}$$

Therefore, absolute *E*_*S*_ can be obtained with an individual level log linear regression: $\mathrm{ln}{\pi }_{k}={b}_{0}+{b}_{1}\mathrm{ln}K+\varepsilon$, where ${\pi }_{k}$ is the number of tokens kept out of a maximum of *K* tokens on a given trial, and absolute ${E}_{S}={b}_{1}$.

### Participants

The experimental protocol was approved by the Institutional Review Board of Virginia Tech. Informed consent and a pre-experiment safety screening questionnaire was obtained from each participant before the experiment started.

## Supplementary information

Supplementary Information.

## Data Availability

The behavioral data that support the findings of this study is available through the Open Science Framework at 10.17605/OSF.IO/FQ2TM.

## Code availability

The analysis scripts that support the findings of this study is available through the Open Science Framework at 10.17605/OSF.IO/FQ2TM.
