# Supplementary material for: Focal stimulation of the temporoparietal junction improves rationality in prosocial decision-making
Source: Sci Rep. 2020 Nov 20;10:20275. doi: 10.1038/s41598-020-76956-9 (PMC7680130; doi:10.1038/s41598-020-76956-9)

Focal Stimulation of the Temporoparietal Junction Improves Rationality in Prosocial Decision-Making

Flora Li1, Sheryl Ball2,3, Xiaomeng Zhang2, and Alec Smith2,3*

^1^Economics Experimental Lab, Nanjing Audit University, Nanjing, China.

^2^Department of Economics, Virginia Tech, Blacksburg, VA, USA.

^3^School of Neuroscience, Virginia Tech, Blacksburg, VA, USA.

*e-mail: [alecsmith@vt.edu](mailto:alecsmith@vt.edu)

September 23, 2020

Supplementary Document

# Supplementary Methods: CES Utility Specification

The charity giving task can be specified as a utility maximization problem using a CES utility function. In this utility maximization problem, $\pi_{k}$ and $\pi_{p}$ represent tokens allocated to the participant and the charity respectively. *K* refers to the maximum possible number of tokens that can be allocated to the participant, and *P* refers to maximum possible number of tokens that can be allocated to the charity.

| $\max_{\pi_{k},\pi_{p}} \left( \alpha\pi_{k}^{\rho}+(1-\alpha)\pi_{p}^{\rho} \right)^{\frac{1}{\rho}} s.t. \frac{\pi_{k}}{K}+\frac{\pi_{p}}{P}\leq1$ | (1.1) |
| --- | --- |

To solve the problem, we first form the associated Lagrangian

| $\mathcal{L}\left( \pi_{k},\pi_{p},\lambda\right)\equiv\left( \alpha\pi_{k}^{\rho}+\left( 1-\alpha\right)\pi_{p}^{\rho} \right)^{\frac{1}{\rho}}+\lambda\left( 1-\frac{\pi_{k}}{K}-\frac{\pi_{p}}{P} \right)$ | (1.2) |
| --- | --- |

Assuming monotonicity in preferences, the budget constraint will hold with equality at the solution. Assuming an interior solution, the Kuhn-Tucker conditions coincide with the ordinary first order Lagrangian conditions and the following equations must hold at the solution values $\pi_{k},\pi_{p},\lambda$:

|  | $\frac{\partial\mathcal{L}}{\partial\pi_{k}}={\alpha\pi_{k}^{\rho-1}\left( \alpha\pi_{k}^{\rho}+\left( 1-\alpha\right)\pi_{p}^{\rho} \right)}^{\frac{1}{\rho}-1}-\frac{\lambda}{K}=0$ | (1.3) |
| --- | --- | --- |
|  | $\frac{\partial\mathcal{L}}{\partial\pi_{p}}={(1-\alpha)\pi_{p}^{\rho-1}\left( \alpha\pi_{k}^{\rho}+\left( 1-\alpha\right)\pi_{p}^{\rho} \right)}^{\frac{1}{\rho}-1}-\frac{\lambda}{P}=0$ | (1.4) |
|  | $\frac{\partial\mathcal{L}}{\partial\lambda}=1-\frac{\pi_{k}}{K}-\frac{\pi_{p}}{P}=0$ | (1.5) |

Combining equations (1.3) and (1.4), we get

| $\lambda=K{\alpha\pi_{k}^{\rho-1}\left( \alpha\pi_{k}^{\rho}+\left( 1-\alpha\right)\pi_{p}^{\rho} \right)}^{\frac{1}{\rho}-1}=P{(1-\alpha)\pi_{p}^{\rho-1}\left( \alpha\pi_{k}^{\rho}+\left( 1-\alpha\right)\pi_{p}^{\rho} \right)}^{\frac{1}{\rho}-1}$ | (1.6) |
| --- | --- |

Solving equation (1.5), we get

| $\pi_{p}=P-\frac{P}{K}\pi_{k}$ | (1.7) |
| --- | --- |

We can get the expenditure function using equations (1.6) and (1.7).

|  | $\begin{matrix} K\alpha\pi_{k}^{\rho-1} & = & P(1-\alpha)\pi_{p}^{\rho-1} \\ \left( \frac{\pi_{p}}{\pi_{k}} \right)^{\rho-1} & = & \frac{K\alpha}{P(1-\alpha)} \\ \frac{\pi_{p}}{\pi_{k}} & = & \left( \frac{K}{P}\cdot\frac{\alpha}{1-\alpha} \right)^{\frac{1}{\rho-1}} \end{matrix}$ | from (1.6) |
| --- | --- | --- |
|  | $\begin{matrix} \frac{P-\frac{P}{K}\pi_{k}}{\pi_{k}} & = & \left( \frac{K}{P}\cdot\frac{\alpha}{1-\alpha} \right)^{\frac{1}{\rho-1}} \\ \frac{P}{\pi_{k}}-\frac{P}{K} & = & \left( \frac{K}{P} \right)^{\frac{1}{\rho-1}}\left( \frac{\alpha}{1-\alpha} \right)^{\frac{1}{\rho-1}} \\ \frac{P}{\pi_{k}} & = & {\frac{P}{K}+\left( \frac{K}{P} \right)}^{\frac{1}{\rho-1}}\left( \frac{\alpha}{1-\alpha} \right)^{\frac{1}{\rho-1}} \end{matrix}$ | from (1.7) |
|  | $\begin{matrix} \pi_{k} & = & \frac{P}{{\frac{P}{K}+\left( \frac{K}{P} \right)}^{\frac{1}{\rho-1}}\left( \frac{\alpha}{1-\alpha} \right)^{\frac{1}{\rho-1}}} \\ \frac{\pi_{k}}{K} & = & \frac{\frac{P}{K}}{{\frac{P}{K}+\left( \frac{K}{P} \right)}^{\frac{1}{\rho-1}}\left( \frac{\alpha}{1-\alpha} \right)^{\frac{1}{\rho-1}}} \\ \frac{\pi_{k}}{K} & = & \frac{\frac{P}{K}\left( \frac{\alpha}{1-\alpha} \right)^{\frac{-1}{\rho-1}}}{\frac{P}{K}\left( \frac{\alpha}{1-\alpha} \right)^{\frac{-1}{\rho-1}}+\left( \frac{K}{P} \right)^{\frac{1}{\rho-1}}} \end{matrix}$ |  |
|  | $\begin{matrix} \frac{\pi_{k}}{K} & = & \frac{\left( \frac{\alpha}{1-\alpha} \right)^{\frac{-1}{\rho-1}}}{\left( \frac{\alpha}{1-\alpha} \right)^{\frac{-1}{\rho-1}}+\left( \frac{K}{P} \right)^{\frac{1}{\rho-1}+1}} \\ \frac{\pi_{k}}{K} & = & \frac{\left( \frac{\alpha}{1-\alpha} \right)^{\frac{1}{1-\rho}}}{\left( \frac{\alpha}{1-\alpha} \right)^{\frac{1}{1-\rho}}+\left( \frac{K}{P} \right)^{\frac{\rho}{\rho-1}}} \end{matrix}$ | (1.8) |

Equation (1.8) is the expenditure function and can be translated into our final structural model.

**Supplementary Methods:** **Hierarchical Random Utility Model (HRUM)**

We used a hierarchical random utility model (HRUM) to estimate the parameters of the CES utility function. We employed a mixed effects approach, where parameter α and ρ were estimated with treatment level fixed effects and subject level random effects. Three tDCS treatment specific regressions were performed using the Stata (Version 15 SE) command menl to allow the variance of residual to differ across treatments. Below we present additional results and statistics from the HRUM estimation.

**Supplementary Figure 1. HRUM estimated Rho and variance of residual.** **P*<.1, ***P*<.05, ****P*<.01. Data in mean±SEM. **a,** ρ parameters shows no difference in mean among treatments (two-sided t-tests: anode vs. cathode *P*=.2169, anode vs. sham *P*=.2003, cathode vs. sham *P*=.9252). **b,** The distribution of the ρ parameter does not differ across treatments (two-sided variance comparison test: anode vs. cathode *P*=.8282, anode vs. sham *P*=.5207, cathode vs. sham *P*=.6703). **c,** The variance of the model residuals is significantly smaller in the anodal treatment than in the cathodal treatment (one-sided t-test: anode vs. cathode *P*<.0001). **d,** Distribution plots for the variance of the residuals shows that the distribution of residuals in the anodal treatment has lower variance than the cathodal distribution.

| **Supplementary Table 1.** HRUMs estimation | | | | | |
| --- | --- | --- | --- | --- | --- |
|  | Alpha (sd) | Rho (sd) | Variance of residuals (sd) | Log likelihood | BIC |
| Cathode | .5958 (.1890) | .0786 (.5143) | .0402 (.0083) | 207.72 | -378.25 |
| Sham | .6462 (.1410) | .0664 (.5542) | .0242 (.0050) | 620.66 | -1204.1 |
| Anode | .5984 (.1309) | .2312 (.4951) | .0265 (.0054) | 533.17 | -1029.1 |

**Supplementary Methods:** **Nonlinear least square (NLS) estimation**

In addition to the HRUM, we also performed a nonlinear least square (NLS) estimation to validate the parameter estimations. Here we assumed that the NLS estimated parameters α and ρ were independent at the subject level. 112 subject specific regressions were performed with Stata (Version 15 SE) command nl. Below, we present results and statistics from the NLS estimation. Overall, the NLS estimation results are consistent with the results from the HRUM.

**Supplementary Figure 2.** **NLS estimated alpha and rho parameters.** **P*<.1, ***P*<.05, ****P*<.01. Data in mean±SEM. **a,** *α* is smaller in the anodal treatment than the sham treatment (one-sided t-test: anode vs. sham *P*=.0957), however is no different between the anodal and cathodal conditions (two-sided t-test: anode vs. cathode *P*=.9677). **b,** Smoothed kernel density of *α* shows that anodal distribution is more concentrated than cathodal distribution (one-sided variance comparison test: anode vs. cathode *P*=.0051). **c,d,** Individuals with ρ<−9 are dropped in the figure due to range limitation in the graph (2 cathode, 1 sham, and 1 anode), but analyses include all participants. **c,** Parameter ρ does not differ across treatments (two-sided t-tests: anode vs. cathode *P*=.3190, anode vs. sham *P*=.3895, cathode vs. sham *P*=.3280). **d,** Anodal stimulation results in a concentration of ρ (one-sided variance comparison test: anode vs. cathode *P*<.0001).

| **Supplementary Table 2.** NLS estimation | | | | | | |
| --- | --- | --- | --- | --- | --- | --- |
|  | Alpha (sd) | Rho (sd) | R squared | Log likelihood | Root mean squared error | BIC |
| Cathode | .6116 (.2501) | -60.95 (352.5) | .9024 | 33.314 | .1547 | -59.035 |
| Sham | .6658 (.1669) | -1.360 (7.154) | .9422 | 33.728 | .1427 | -59.631 |
| Anode | .6137 (.1582) | -.2503 (2.149) | .9343 | 31.147 | .1481 | -54.470 |

# Supplementary Figures

**Supplementary Figure 3. Participants’ self-reported tDCS sensations.** Data in mean±95%CI. Participants self-report tDCS sensation levels in the post-experiment survey. 1 = Absent, 2 = Mild, 3 = Moderate, and 4 = Severe. Sham participants report more Scalp Pain than anodal participants (ranksum test: *P*=.0322). Anodal participants report marginally less Mood Change than cathodal participants (ranksum test: *P*=.0787). These two results are surprising due to the direction of the effect. Cathodal participants report marginally more Itching than anodal (ranksum test: *P*=.0856) and sham (ranksum test: *P*=.0982) participants. For all the other ratings, participants report very similar sensation levels across three treatments.

**Supplementary Figure 4. Illustration of CCEI.** CCEI $\in[0,1]$ measures how far budget constraints must be shifted to avoid a GARP violation. Both X and Y are available under budget B1 and B2. Choosing X under B1 and choosing Y under B2 violates both WARP and GARP. To eliminate the violation we can shift B2 to B2’ such that Y is no longer available under B2. CCEI=1 represents no GARP violation, and CCEI=0 represents the most severe GARP violation.

**Supplementary Figure 5. Monotonicity violations.** **P*<.1, ***P*<.05, and ****P*<.01. Data in mean±SEM. **a,** Monotonicity: for X and Y available under budget B, if X is strictly greater than Y, then X is preferred to Y. For monotonicity to hold, an individual chooses allocations on the budget line. **b,** Anodal participants have fewer Monotonicity violations (anode vs. cathode one-sided t-test: *P*=.0981). **c,** Absolute distance to budget line measures Monotonicity violation severity. Violations of Monotonicity in the anodal treatment is the least severe (anode vs. cathode one-sided t-test: *P*=.0742). **d,** Vertical and horizontal distances to budget line is the shortest in the anodal treatment (anode vs. cathode one-sided t-test: vertical *P*=.0704, horizontal *P*=.0639). We do not observe hemifield neglect caused by tDCS over rTPJ; two-sided t-tests of the difference in means (vertical vs. horizontal distance) cannot rejects the null hypothesis of no difference (anode *P*=.8714, cathode *P*=.8421, sham *P*=.7722).

**Supplementary Figure 6. WARP violations.** **P*<.1, ***P*<.05, and ****P*<.01. Data in mean±SEM. **a,** WARP: If X is directly revealed preferred to Y, then we do not have Y directly revealed preferred to X. WARP implies that if an individual chooses X over Y when the budget line is B1, she cannot choose Y over X when the budget line is B2. **b,** There are fewest WARP violations in the anodal treatment; a t-test of the difference in means (anode vs. cathode) rejects the null hypothesis of no difference (*P*=.0182).

**Supplementary Figure 7. Rationality violations over time.** Data in mean±SEM. For all three rationality measures, we observe least anodal violations starting from the beginning of the experiment (anode vs. cathode first 10 rounds one-sided t-tests: Monotonicity *P*=.0361, WARP *P*=.0036, GARP *P*=.0029). Among all treatments and all rationality measures, there is no difference in violation frequency comparing first vs. last 10 rounds; two-sided t-tests of the difference in means cannot reject the null hypothesis of no difference (Monotonicity: anode *P*=.6349, cathode *P*=.2385, sham *P*=.1510; WARP: anode *P*=.8913, cathode *P*=.5600, sham *P*=.3907; GARP: anode *P*=.9640, cathode *P*=.6973, sham *P*=.2997).

**Supplementary Figure 8. Welfare, budget, and efficiency.** **P*<.1, ***P*<.05, and ****P*<.01. Data in mean±SEM. **a,** Anodal participants achieves highest welfare among all treatments (one-sided t-test: anode<cathode *P*=.0324; anode<sham *P*=.0331). **b,** Budgets are relative same across treatments, except that anode has marginally larger budget than sham (one-sided t-test: *P*=.0823). **c,** Anodal participants are marginally more efficient than cathodal participants (one-sided t-test: *P*=.0591).

**Supplementary Figure 9. Reaction times over time.** Data in mean±SEM. **a,** Reaction time over every 10 rounds shows reaction time decreases over time (first vs. last 10 rounds one-sided t-test: anode *P*=.0052, cathode *P*=.0001, sham *P*=.0006; 1st vs. 2nd half ranksum test: anode *P*=.0430, cathode *P*=.0119, sham *P*=.0137). **b,** Subject Level reaction time are not different across treatments (two-sided t-test: anode vs. cathode *P*=.9023, anode vs. sham *P*=.4548, cathode vs. sham *P*=.3136).

# Representative Sample Decisions

| **** | **** |  |
| --- | --- | --- |
| **a** # 36, *α* = .026, *ρ* = −9.550 | **b** # 59, *α* = .619, *ρ* = −11.666 | **c** # 4, *α* = .801, *ρ* = −41.380 |
| **Supplementary Figure 10. Rawlsians with *ρ* → −∞** | | |
|  |  |  |
| **a** # 24, *α* = .354, *ρ* = .092 | **b** # 19, *α* = .546, *ρ* = .043 | **c** # 51, *α* = .931, *ρ* = −.047 |
| **Supplementary Figure 11. Cobb-Douglas with *ρ* → 0** | | |
| **** | **** | **** |
| **a** # 99, *α* = .450, *ρ* = .997 | **b** # 95, *α* = .509, ρ = .990 | **c** # 94, *α* = .726, *ρ* = .827 |
| **Supplementary Figure 12. Utilitarians with *ρ* → 1** | | |

Supplementary Figures 10, 11, and 12 show 50 decisions from 9 participants. The orange dashed line represents an equal split of the tokens. Supplementary Figure 10 shows individuals with Rawlsian utility functions where *ρ*→−∞. These individuals have L-shaped or Leontief utility. Supplementary Figure 11 shows individuals who have Cobb-Doulas utility functions, which are bowed in and smooth. Supplementary Figure 12 shows individuals with Utilitarian utility functions, which is represented with a straight line, therefore, those individuals prefer allocations where the budget line touches either of the axes. Figures 10a,11a,12a show allocations from generous individuals, Figures 10b,11b,12b show allocations from individuals with strong preferences for fairness, and Figures 10c,11c,12c show allocations from selfish individuals.

**Instructions**

The experiment instructions are reproduced below. The original instructions have black backgrounds. For ease of reading, the colors are inverted here. The original budget lines were displayed in green, here they appear pink.


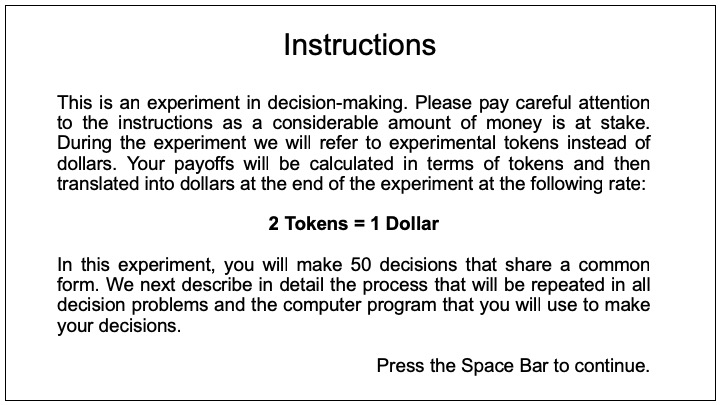


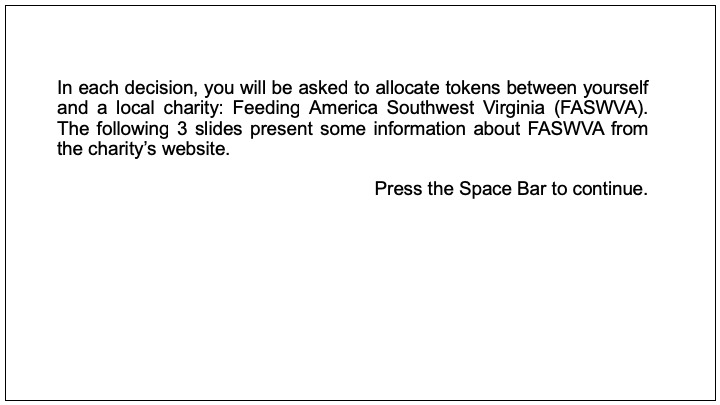


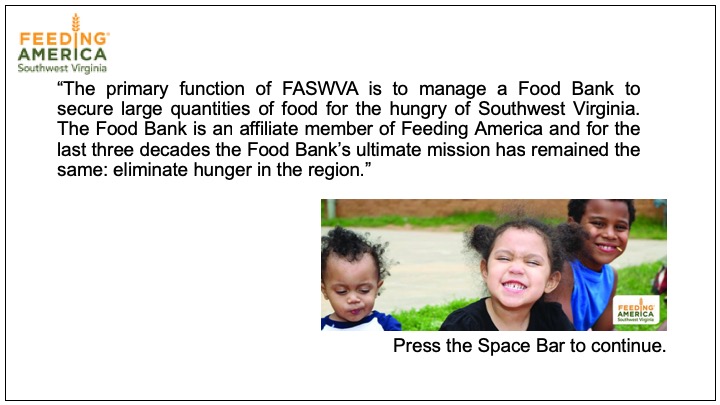


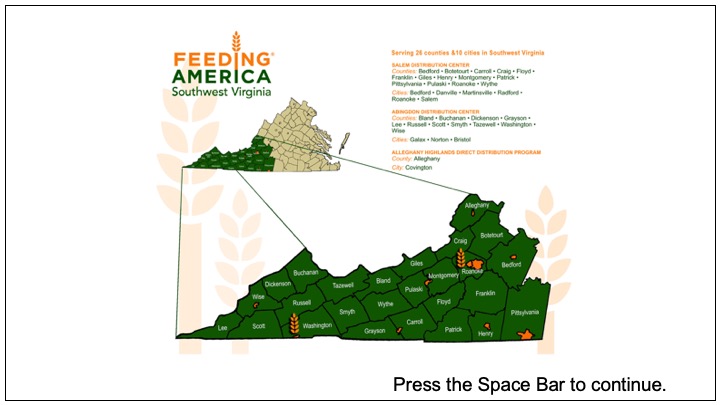


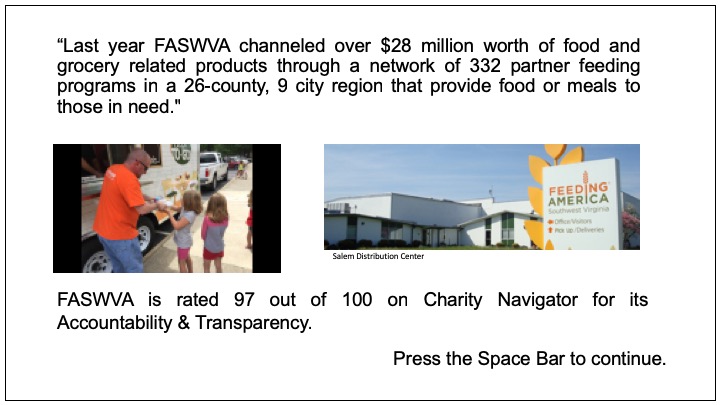


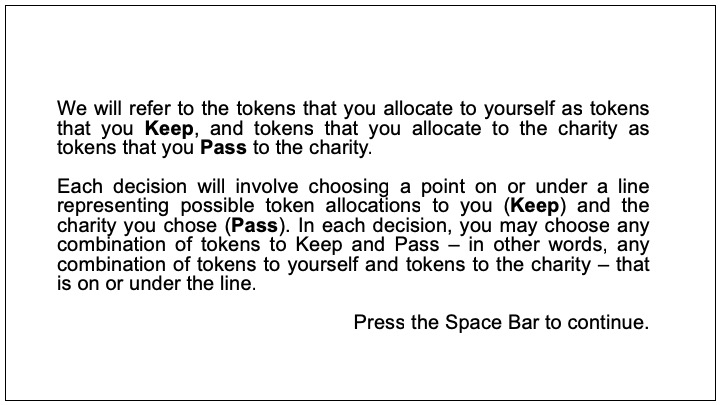


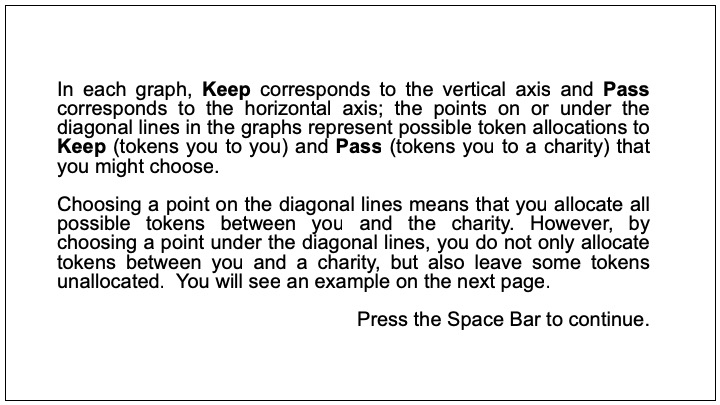


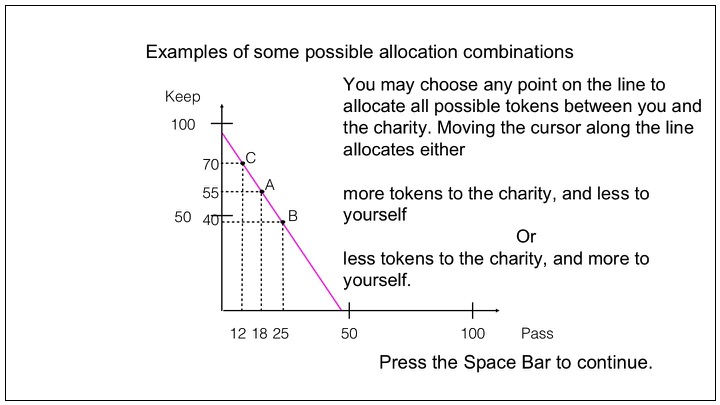


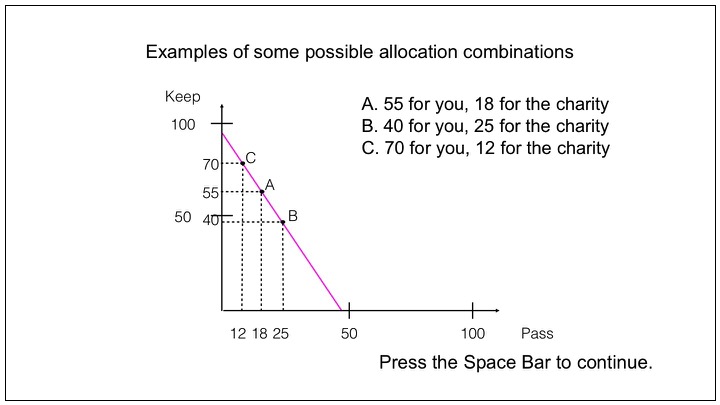


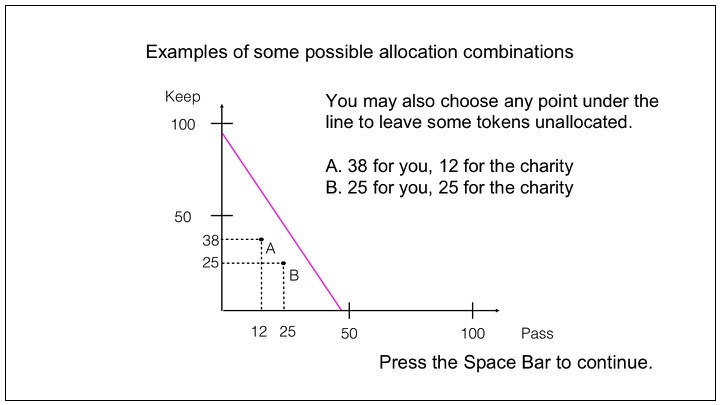


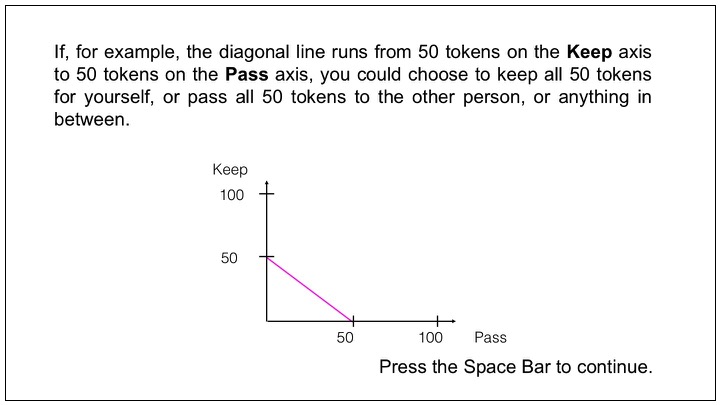


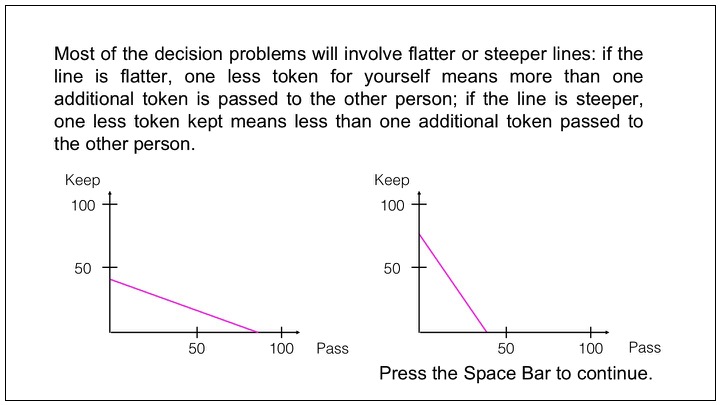


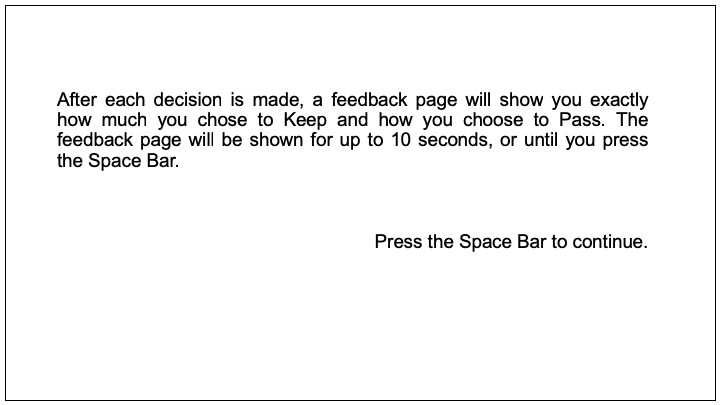


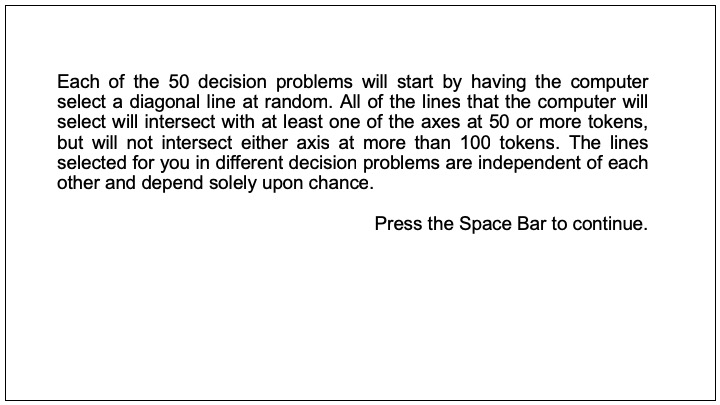


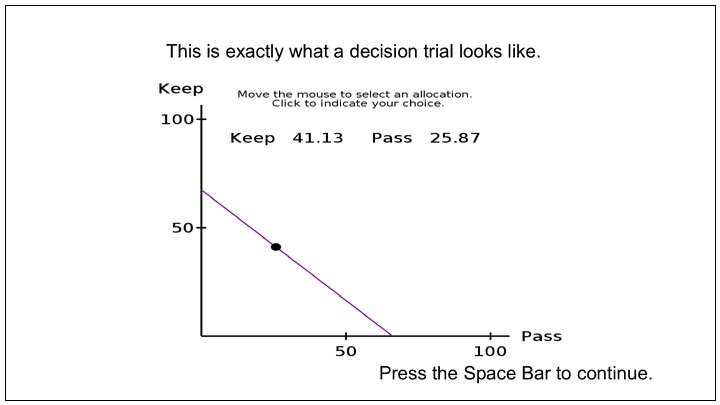


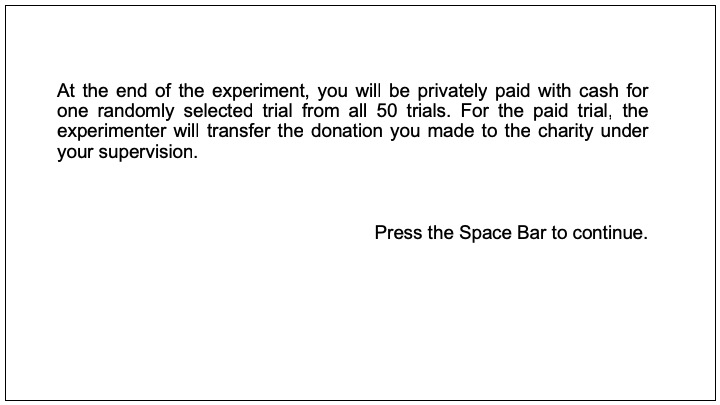


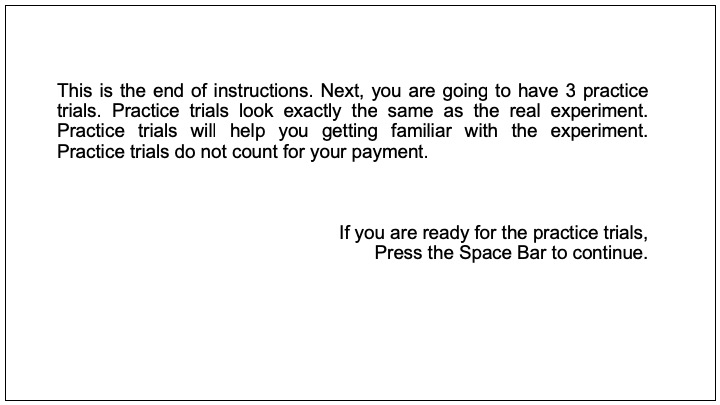

Supplement: Supplementary file 1 — Supplementary Information. [file 41598_2020_76956_MOESM1_ESM.docx]
